# Effects of FER1L4-miR-106a-5p/miR-372-5p-E2F1 regulatory axis on drug resistance in liver cancer chemotherapy

**DOI:** 10.1016/j.omtn.2021.02.006

**Published:** 2021-02-10

**Authors:** Xu Wang, Yunfei Chen, Ke Dong, Yujing Ma, Qizhi Jin, Shujun Yin, Xiaoshi Zhu, Shan Wang

**Affiliations:** 1 The Second Ward of Hepatobiliary Surgery, Sichuan Provincial People’s Hospital, University of Electronic Science and Technology of China and Chinese Academy of Sciences Sichuan Translational Medicine Research Hospital, Chengdu 610072, P.R. China; 2 Organ Transplant Center, Sichuan Provincial People’s Hospital, University of Electronic Science and Technology of China and Chinese Academy of Sciences Sichuan Translational Medicine Research Hospital, Chengdu 610072, P.R. China; 3 The Third Ward of Hepatobiliary Surgery, Sichuan Provincial People’s Hospital, University of Electronic Science and Technology of China and Chinese Academy of Sciences Sichuan Translational Medicine Research Hospital, Chengdu 610072, P.R. China; 4 Pediatric Intensive Care Unit, Sichuan Provincial People’s Hospital, University of Electronic Science and Technology of China and Chinese Academy of Sciences Sichuan Translational Medicine Research Hospital, Chengdu 610072, P.R. China; 5 Ultrasound in Cardiac Electrophysiology and Biomechanics Key Laboratory of Sichuan Province, Sichuan Provincial People’s Hospital, University of Electronic Science and Technology of China and Chinese Academy of Sciences Sichuan Translational Medicine Research Hospital, Chengdu 610072, P.R. China

**Keywords:** FER1L4, miR-106a-5p, miR-372-5p, E2F1, NF-κB, liver cancer, chemotherapy resistance

## Abstract

Liver cancer presents a challenge in today’s healthcare system. This study aimed at investigating the effects of Fer-1 like family member 4 (FER1L4) on chemotherapy resistance and liver cancer development by using clinically collected liver cancer tissues and commercially purchased human liver cancer cisplatin-resistant cell line HUH-7/DDP. Bioinformatics analysis, dual luciferase reporter gene assay, and RNA pull-down were applied to predict and verify the possible binding relationships. The expressions of FER1L4, E2F transcription factor 1 (E2F1), microRNA-106a-5p (miR-106a-5p), or miR-372-5p were altered in the cells, followed by flow cytometry, Cell Counting Kit-8 (CCK-8), and Transwell assays to evaluate apoptotic, proliferative, and invasive abilities *in vitro* and nude mice xenografts to observe tumor growth *in vivo*. FER1L4 was highly expressed and miR-106-5p and miR-372-5p were poorly expressed in tumor cells and tissues. FER1L4 knockdown or the overexpression of miR-106-5p and miR-372-5p inhibited the cancerous cell proliferation and invasion while promoting apoptosis. FERIL4 silencing increased the miR-106-5p/miR-372-5p expression to inhibit the E2F1-activated nuclear factor κB (NF-κB) pathway. Besides, overexpressing FER1L4 led to an increased tumor growth in nude mice, which was reversed by the NF-κB inhibitor pyrollidine dithiocarbamate (PDTC). In conclusion, the results indicated that FER1L4 could inhibit the expression of miR-106a-5p/miR-372-5p, to activate E2F1-mediated NF-κB pathway, leading to drug resistance in liver cancer.

## Graphical Abstract

**Figure:**
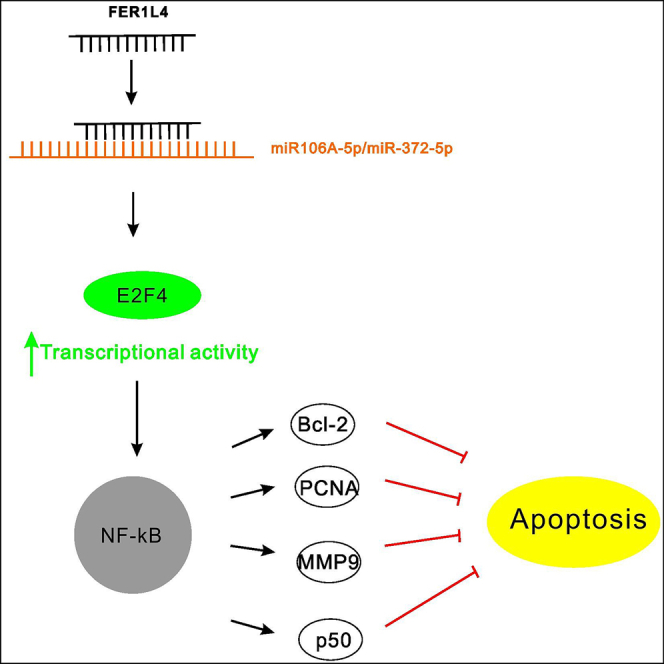

## Introduction

The incidence of liver cancer has increased by 4.6% from 2005 to 2015, although the incidence of cancers in general is in the downward trend.^1^ This alarming news put liver cancer as the fourth most deadly cancer in the world.^2^^,^^3^ In China, it was estimated that there were 370,000 new cases of liver cancer in 2015, which was approximately 17.64 per 100,000 people.^4^ There are wide regional differences in the incidence of liver cancer around the world, with the majority of liver cancer cases occurring in low to medium socioeconomic societies.^5^ Similar to this, rural areas appear to have a high incidence of liver cancer in China.^4^ Heavy alcohol consumption, hepatitis B and C infections, and exposure to dietary toxins may be a few important risk factors for the development of liver cancer in China,^6^^,^^7^ with hepatitis B and C infections accounting for 80% of all hepatocellular carcinoma cases.^8^^,^^9^ Despite improvements in the early detection of liver cancer,^10^ the increasing incidence may be attributed to the incomplete understanding of its pathology and drug resistance.^11^^,^^12^ Therefore, we investigated the pathway that was involved in the progression of liver cancer in a drug-resistant strain.

Previous studies have found a close relationship between the deregulation of long non-coding RNAs (lncRNAs), a new class of regulatory non-coding RNA, and human tumor progression. For example, lncRNA Fer-1-like family member 4 (FER1L4) has been reported to be involved in the development of gastric cancer.^13^ Besides, FER1L4 can be used as a prognostic biomarker, and its low expression was identified as an important independent predictor of poor survival in endometrial cancer.^14^^,^^15^ FER1L4 is also involved in esophageal squamous cells carcinogenesis and development.^16^ In this study, we sought to examine the potential role of FER1L4 in the progression of liver cancer. Previous studies have suggested the possible involvement of FER1L4 in the progression of colon cancer by regulating microRNA-106a-5p (miR-106a-5p).^17^ Moreover, FER1L4/miR-372/E2F transcription factor 1 (E2F1) is a competing endogenous RNA (ceRNA) system that regulates the proliferation and cell-cycle distribution in glioma cells.^18^ The expression level of miR-106a is inversely proportional to its target gene E2F1, which was demonstrated to inhibit the growth of glioma cells by targeting E2F1.^19^^,^^20^ Additionally, E2F1 is a direct target for miR-372-5p,^21^ which is also a key determinant of the G1/S phase transition in the cell cycle that activates gene transcription encoding proteins required for DNA replication. E2F1 is a transcriptional activator of nuclear factor κB (NF-κB) target genes^22^ and promotes apoptosis by inhibiting NF-κB activity.^23^ Based on the above results, we determined the targeting relationships and effects of miR-106a, miR-372-5p, E2F1, and NF-κB as downstream signaling molecules in drug-resistant liver cancer by using cell and animal models.

## Results

### FER1L4 expression is increased in liver cancer tissues and cell lines

**Figure 1:**
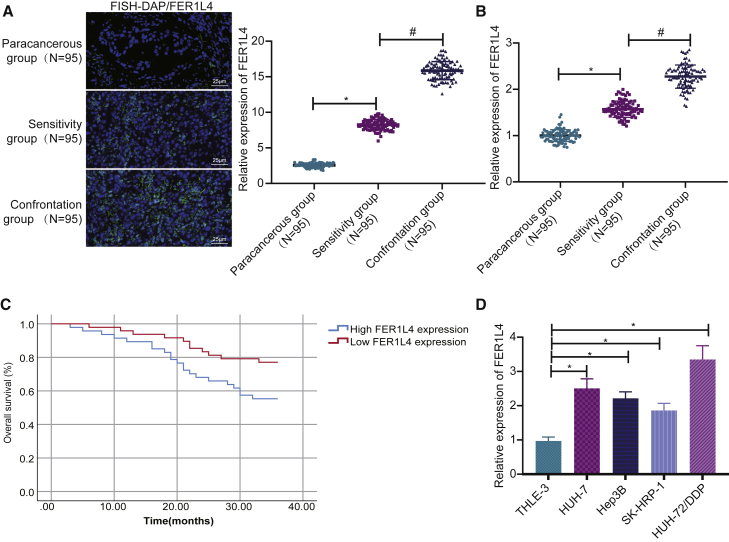
The expression of FER1L4 is increased in liver cancer (A) FER1L4 expression in 95 cases of liver cancer patients determined by FISH. (B) FER1L4 expression in 95 cases of liver cancer cisplatin drug-sensitive and -resistance groups. (C) FER1L4 expression and patient survival determined by Kaplan-Meier method. (D) FER1L4 expression in cancer cell lines. Data are expressed by the mean ± SD. One-way ANOVA and post hoc Tukey’s test were used for comparisons between multiple groups. Kaplan-Meier method was used to calculate patient survival.

In this study, fluorescence *in situ* hybridization (FISH) and quantitative reverse transcriptase polymerase chain reaction (qRT-PCR) assays were employed to explore the relationship between FER1L4 and the progression of liver cancer and the resistance of liver cancer cells. The results revealed that the expression of FER1L4 in liver cancer tissues was significantly increased compared with that of the adjacent tissues (Figure 1A). The expression of FER1L4 in the drug-resistant group was higher than that in the drug-sensitive group (Figure 1B). In addition, a high expression of FER1L4 was found to be associated with a poorer prognosis (Figure 1C). Moreover, the expression of FER1L4 in liver cancer cell lines SK-HEP-1, Hep3B, and HUH-7 and HUH-7/DDP-resistant cell line was significantly increased compared with that of the normal liver cell line THLE-3, and among them, HUH-7/DDP-resistant strains had the highest expression of FER1L4 (Figure 1D). Therefore, HUH-7/DDP-resistant cells were used in subsequent experiments.

### FER1L4 knockdown inhibits the growth of liver cancer cells *in vitro*

**Figure 2:**
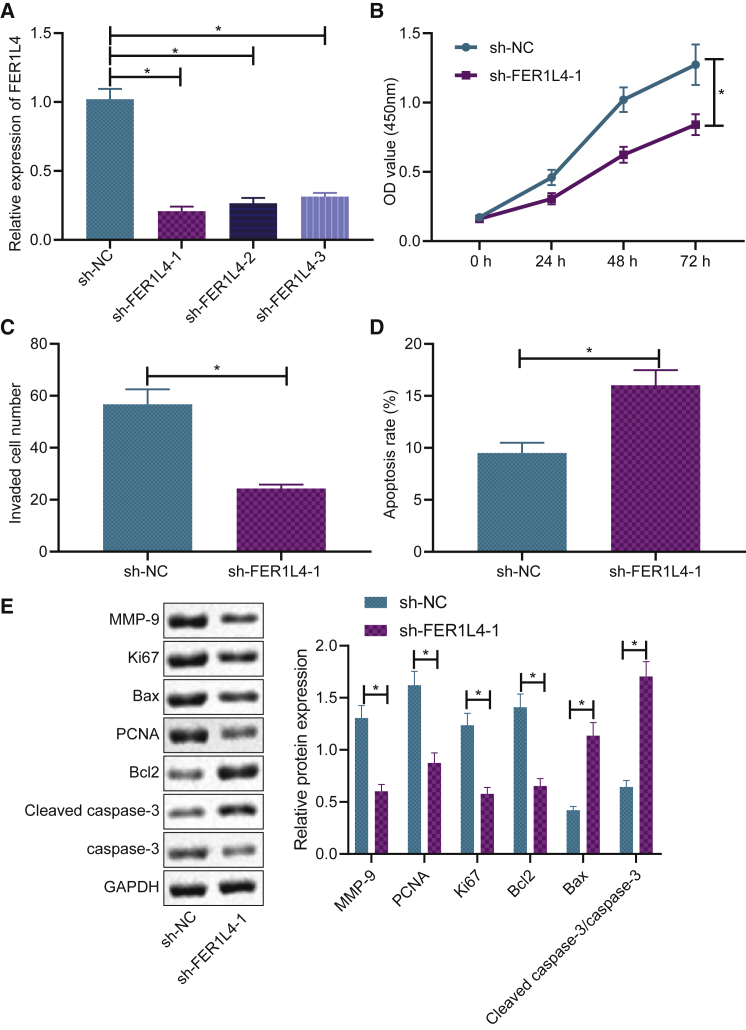
FER1L4 knockdown inhibits liver cancer cell growth (A) Effectiveness of FER1L4 knockdown. (B) Cell proliferation determined by CCK-8 assay. (C) Cell invasion determined by Transwell assay. (D) Cell apoptosis determined by flow cytometry. (E) Protein expressions of Bcl-2, Bax, Ki67, and PCNA and expression levels of MMP9. Data are expressed by the mean ± SD. Paired t test was used to compare adjacent tissues with cancer tissues. One-way ANOVA and post hoc Tukey’s test were used for comparisons between multiple groups.

In order to investigate the function that FER1L4 had on the proliferation, invasion, and apoptotic capabilities of the HUH-7/DDP-resistant cells, three short hairpin RNAs (shRNAs) against FER1L4 (sh-FER1L4-1, sh-FER1L4-2, and sh-FER1L4-3) were used to knock down the expression of FER1L4 in the cells. As illustrated in Figure 2A, the three shRNAs had successfully reduced the expression of FER1L4 in the HUH-7/DDP-resistant strains, while the sh-FER1L4-1 group had the lowest expression of FER1L4, which was selected for subsequent experiments (Figure 2A). Subsequently, the cell proliferative, invasive, and apoptotic capabilities were evaluated by Cell Counting Kit-8 (CCK-8), Transwell, and flow cytometry assays. FER1L4 knockdown was observed to inhibit cell proliferation (Figure 2B) and cell invasion (Figure 2C) but promote cell apoptosis (Figure 2D). In addition, based on western blot analysis, it was revealed that the silencing of FER1L4 decreased the protein level of proliferation-related genes Ki67 and PNCA and invasion-related gene MMP9, as well as apoptosis-related gene Bcl2, while the expressions of Bax protein and cleaved caspase-3 were increased (Figure 2E). These results suggested that FER1L4 knockdown may exert an inhibitory role in the progression of liver cancer, as evident by promoting cell apoptotic ability and reducing proliferative and invasive capabilities.

### miR-106a-5p and miR-372-5p expressions are decreased in liver cancer tissues and cells to affect the cellular processes

**Figure 3:**
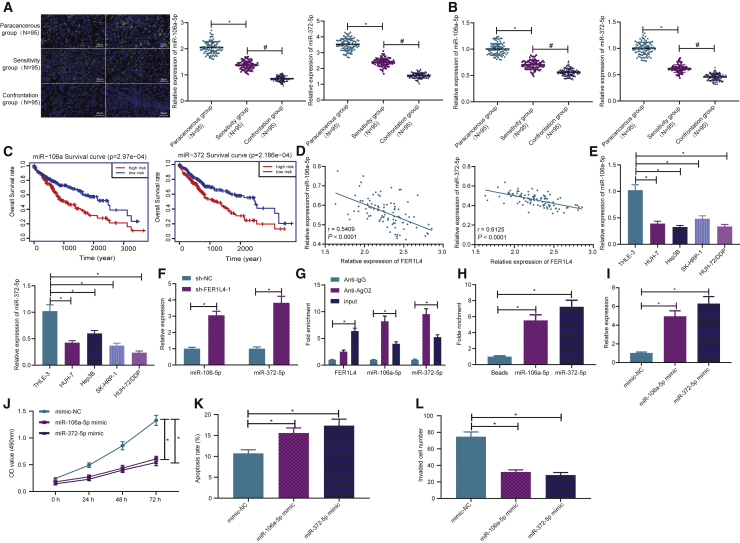
Expressions of miR-106a-5p and miR-372-5p are decreased in liver cancer and inhibit liver cancer cells (A) Expressions of miR-106a-5p and miR-372-5p in 95 cases of liver cancer patients determined by FISH. (B) Expressions of miR-106a-5p and miR-372-5p in 95 cases of liver cancer drug sensitivity and resistance groups. (C) Bioinformatics analysis on the expressions of miR-106a-5p and miR-372-5p and total survival. (D) Correlation between miR-106a-5p, miR-372-5p, and FER1L4 expression determined by Pearson. (E) Expressions of miR-106a-5p and miR-372-5p in liver cancer cells; ∗p < 0.05 versus THLE-3 cells. (F) Effects of FER1L4 knockdown on miR-106a-5p and miR-372-5p expressions in HUH-7/DDP cells. (G) Binding relationship between FER1L4, miR-372-5p, and miR-106a-5p determined by RIP; ∗p < 0.05 versus anti-AigG. (H) Expressions of miR-372-5p and miR-106a-5p in biotinylated FER1L4 determined by RNA pull-down. (I) Effects of miR-106a-5p and miR-372-5p mimic on the expressions of miR-106a-5p and miR-372-5p. (J) Cell proliferation determined by CCK-8 assay. (K) Apoptosis determined by flow cytometry. (L) Cell invasion determined by Transwell assay. Data are expressed by the mean ± SD. Paired t test was used to compare adjacent tissues with cancer tissues. One-way ANOVA and post hoc Tukey’s test were used for comparisons between multiple groups.

For the purpose of investigating the relationship between FER1L4 and miR-106a-5p and between FER1L4 and miR-372-5p, we initially detected the expressions of miR-106a-5p and miR-372-5p in patients with liver cancer. As depicted in Figures 3A and 3B, the expressions of miR-106a-5p and miR-372-5p were reduced in liver cancer tissues relative to that of adjacent tissues, while the lowest expression was found in cisplatin-resistant drug group. Following this, bioinformatics analysis indicated that, patients with a lower expression of miR-106a-5p or miR-372-5p showed lower overall survival (OS) than those with a higher expression of miR-106a-5p or miR-372-5p (Figure 3C), suggesting that low expressions of miR-106a-5p and miR-372-5p were related to a poorer prognosis and survival of patients with liver cancer. Besides, it was also revealed that the expressions of miR-106a-5p and miR-372-5p were negatively related to the expression of FER1L4 (Figure 3D). According to qRT-PCR results shown, the expressions of miR-106a-5p and miR-372-5p in SK-HEP-1, Hep3B, HUH-7, and HUH-7/DDP cells were lower when compared to that of normal liver cells THLE-3, with HUH-7/DDP cells having the lowest expression (Figure 3E). After knocking down FER1L4 expression, the expressions of miR-106-5p and miR-372-5p were increased in the liver cancer cisplatin-resistant strains (Figure 3F).

miRNA exists in the cytoplasm as a component of RNA-induced silencing complex (RISC), and Ago2 is known as a key component of RISC, which is necessary for miRNA-mediated gene silencing. Therefore, in this study, anti-Ago2 antibodies were used in RNA immunoprecipitation (RIP) experiments in liver cancer cisplatin-resistant cell extracts to determine whether FER1L4 and miR-106-5p/miR-372-5p belong to the same RISC. We found that Ago2 miRNA was enriched in the FER1L4 group relative to that in the immunoglobulin G (IgG) group (Figure 3G). Also, miR-106-5p/miR-372-5p was presented in the same precipitate (Figure 3G). The RNA pull-down assay with qRT-PCR was employed, which detected the expression of miR-106-5p/miR-372-5p in RNA-RNA complex, to reveal the direct binding sites of FER1L4 to miR-106-5p and miR-372-5p (Figure 3H).

Transfection efficiency was subsequently tested after transfection of miR-106a-5p mimic and miR-372-5p mimic, suggesting successful transfection (Figure 3I). The proliferative, apoptotic, and invasive capabilities of the cells were then measured by CCK-8, flow cytometry, and Transwell assays. It was observed that miR-106a-5p and miR-372-5p mimic inhibited cell proliferation (Figure 3J), increased apoptosis (Figure 3K), and reduced invasion capabilities (Figure 3L).

In brief, the above results indicated that in human liver cancer cisplatin-resistant cell line HUH-7/DDP, FER1L4 was discovered to bind and regulate miR-106a-5p and miR-372-5p. The overexpression of miR-106a-5p and miR-372-5p inhibited proliferation and invasion capabilities and promoted apoptosis in human liver cancer cells.

### miR-106a-5p and miR-372-5p inhibits development of liver cancer cells by targeting E2F1

**Figure 4:**
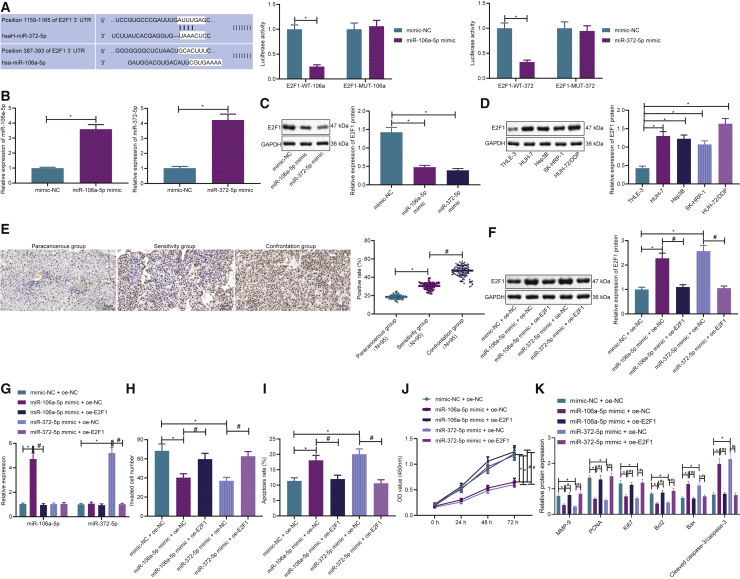
miR-106a-5p and miR-372-5p inhibit liver cancer cells by targeting E2F1 (A) Bioinformatics analysis of targeting between miR-106a-5p, miR-372-5p, and E2F1. Binding relationships were verified by dual luciferase reporter assay. (B) Expressions of miR-106a-5p and miR-372-5p in HUH-7/DDP cells after transfection. (C) Expression of E2F1 after miR-106a-5p and miR-372-5p transfection determined by western blotting. (D) Expression of E2F1 in liver cancer cells. (E) Expression of E2F1 in liver cancer tissues determined by immunohistochemistry. (F) Expression of E2F1 in different experimental groups determined by western blotting. (G) Expressions of miR-106a-5p and miR-372-5p in different experimental groups. (H) Cell invasion determined by Transwell assay. (I) Cell apoptosis determined by flow cytometry. (J) Cell proliferation determined by CCK-8 assay. (K) Expressions of E2F1, Bcl-2, Bax, Ki67, PCNA, and MMP9; data are expressed by the mean ± SD. Unpaired t test was used for the comparison between two groups. Repeated measures ANOVA was used for tumor volume comparison at different time points.

In order to survey the interaction between miR-106a-5p/miR-372-5p and E2F1, bioinformatics analysis was employed using TargetScan and MicroRNA.org online databases, which predicted that miR-106a-5p and miR-372-5p targeted E2F1 and revealed binding sites. For further validation purposes, we performed a dual luciferase reporter gene assay, which indicated a lower luciferase activity after the co-expression of miR-106a-5p mimic/miR-372-5p mimic and E2F1-wild-type (WT), compared with that of the co-expression of miR-106a-5p mimic/miR-372-5p mimic and negative control (NC) plasmid, while no significant difference was observed after the co-expression of miR-106a-5p mimic/miR-372-5p mimic and E2F1 mutant (MUT; Figure 4A). As a result, a direct targeting relationship exists between miR-106a-5p and miR-372-5p and E2F1.

In addition, qRT-PCR revealed that miR-106a-5p mimic and miR-372-5p mimic had increased the expressions of miR-106a-5p and miR-372-5p, respectively (Figure 4B), while the decreased expression of E2F1 was demonstrated by western blot analysis (Figure 4C), which further suggested the regulation of miR-106a-5p and miR-372-5p on the expression of E2F1. Western blot analysis suggested that the expression of E2F1 was higher in all liver cancer cell lines relative to that of normal liver cells, in which cisplatin-resistant cell line HUH-7/DDP was the highest (Figure 4D). In addition, as shown in Figure 4E, the results of the immunohistochemistry assay revealed that the expression of E2F1 in liver cancer tissues was significantly increased compared with that of adjacent tissues and was also the highest in the cisplatin-resistant drug group.

Afterward, we further evaluated whether miR-106a-5p and miR-372-5p regulates cell functions through its modulation on E2F1. The results demonstrated that miR-106a-5p mimic and miR-372-5p mimic reduced the expressions of E2F1, which was increased by the addition of E2F1 overexpression (Figure 4F). miR-106a-5p mimic and miR-372-5p mimic increased the expressions of miR-106a-5p and miR-372-5p (Figure 4G). miR-106a-5p mimic and miR-372-5p mimic reduced cell invasion (Figure 4H), promoted cell apoptosis (Figure 4I), and reduced proliferation (Figure 4J). These effects were reversed by the addition of E2F1 overexpression. miR-106a-5p mimic and miR-372-5p mimic reduced the expressions of Bcl-2, Ki67, PCNA, and MMP9, while increasing the expressions of Bax and cleaved caspase-3 (Figure 4K), which were reversed by overexpressing E2F1. The above results indicated that miR-106a-5p and miR-372-5p mimic inhibited the cell proliferation and invasion capabilities and promoted the cell apoptosis by targeting E2F1 in human liver cancer cisplatin-resistant cell line HUH-7/DDP.

### FER1L4 promoted liver cancer progression through the miR-106a-5p/miR-372-5p-E2F1 signaling

**Figure 5:**
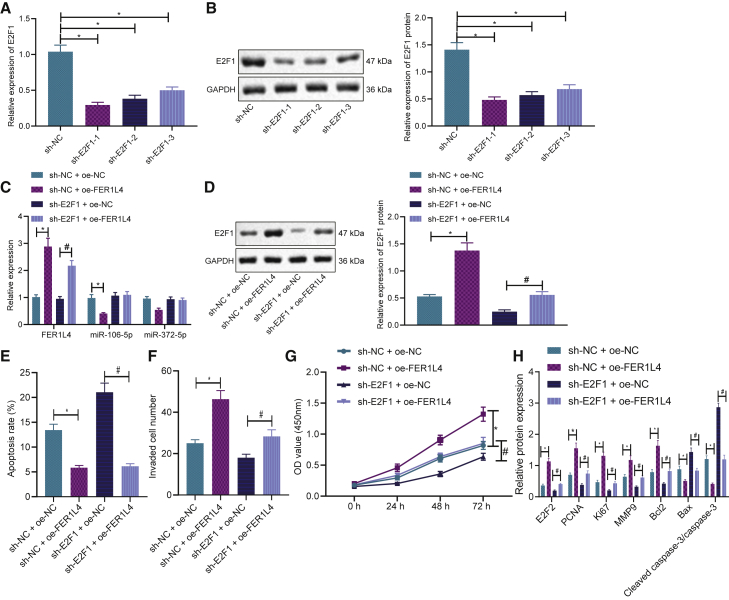
FER1L4 promoted liver cancer through the miR-106a-5p/miR-372-5p-E2F1 signaling (A) Effectiveness of E2F1 knockdown by qRT-PCR. (B) Effectiveness of E2F1 knockdown by western blotting. (C) mRNA expressions of FER1L4, miR-106a-5p, and miR-372-5p in different experimental groups. (D) E2F1 protein expression in different experimental groups. (E) Cell apoptosis determined by flow cytometry. (F) Cell invasion determined by Transwell assay. (G) Cell proliferation determined by CCK-8 assay. (H) Expressions of E2F1, Bcl-2, Ki67, PCNA, MMP9, and Bax protein in different experimental groups. Data are expressed by the mean ± SD. One-way ANOVA and post hoc Tukey’s test were used for comparisons between multiple groups. A two-way ANOVA was used to compare values at different time points.

The above-mentioned results uncovered that FER1L4 could bind to miR-106a-5p and miR-372-5p and regulate their expressions, which further targets E2F1 to regulate liver cancer cell processes. To further explore the regulatory mechanism of the FER1L4-miR-106-5p/miR-372-5p-E2F1 axis, we first used three shRNAs against E2F1 to knock down the expression of E2F1 in the cells, and sh-E2F1-1 was found to have the best silencing efficiency, which was selected for subsequent experiments (Figures 5A and 5B). According to qRT-PCR and western blot analyses, overexpressing FER1L4 levels increased the expressions of FER1L4 and E2F1, while reducing that of miR-106a-5p and miR-372-5p (Figures 5C and 5D). E2F1 knockdown reduced the expression of E2F1 but had no effect on that of miR-106a-5p and miR-372-5p. Moreover, cell functions were evaluated by flow cytometry, Transwell, and CCK-8. Overexpressing FER1L4 levels was observed to suppress cell apoptosis (Figure 5E), promote invasion (Figure 5F), and enhance proliferation capabilities (Figure 5G) when compared to NC or E2F1 knockdown alone. Finally, Figure 5H illustrates the measurement results of genes related to proliferation, invasion, and apoptosis. Overexpressing FER1L4 increased the expressions of E2F1, Bcl-2, Ki67, PCNA, and MMP9 while reducing the expressions of Bax and cleaved caspase-3 when compared to that of NC or E2F1 knockdown alone (Figure 5H). These results showed that FER1L4 was bound to miR-106-5p and miR-372-5p in order to block the targeted inhibition of E2F1, thereby promoting the progression of liver cancer.

### E2F1 promotes drug-resistant liver cancer cell growth by activating the NF-κB pathway

**Figure 6:**
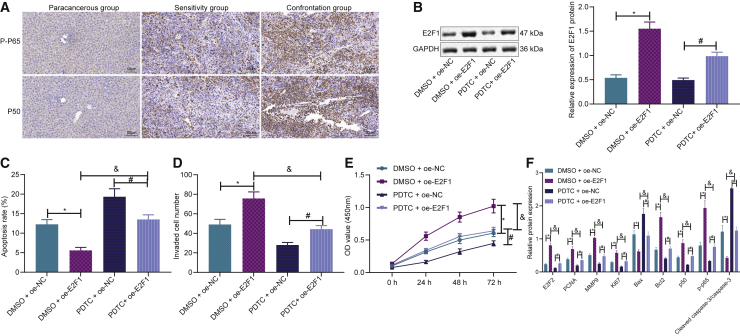
E2F1 promotes drug-resistant liver cancer cells by activating the NF-κB pathway (A) Expressions of p50 and p-p65 in the NF-κB pathway determined by immunohistochemistry. (B) Expressions of E2F1 in different experimental groups. (C) Cell apoptosis by flow cytometry. (D) Cell invasion determined by Transwell assay. (E) Cell proliferation by CCK-8 assay. (F) E2F1, p50, p-p65, Bcl-2, Bax, Ki67, PCNA, and MMP9 protein expressions. Data are expressed by the mean ± SD. One-way ANOVA and post hoc Tukey’s test were used for comparisons between multiple groups. Two-way ANOVA was used to compare values at different time points.

Furthermore, more attention was focused on whether the NF-κB pathway is involved in the modulation of E2F1 on liver cancer cells. Immunohistochemistry revealed that the expressions of p50 and p-p65 were significantly increased in liver cancer tissues (Figure 6A). The NF-κB pathway inhibitor PDTC and E2F1-overexpressed plasmids were then used to transfect the cells. It was found that overexpressing E2F1 increased the expression of E2F1 when compared to that of NC or NF-κB pathway inhibitor PDTC alone (Figure 6B). Moreover, cell function experiments revealed that overexpressing E2F1 levels reduced cell apoptosis (Figure 6C) and increased invasion (Figure 6D) and proliferation capabilities (Figure 6E), which were reversed by the addition of PDTC. In addition, overexpressing E2F1 levels increased the expressions of E2F1, p50, p-p65, Bcl-2, Ki67, PCNA, and MMP9, while decreasing the expression of Bax (Figure 6F). These effects were inhibited by the addition of PDTC. The above results showed that E2F1 promoted liver cancer cisplatin-resistant drug strains by activating the NF-κB pathway.

### FER1L4 affects chemotherapy resistance in tumor-bearing mice by regulating NF-κB pathway

**Figure 7:**
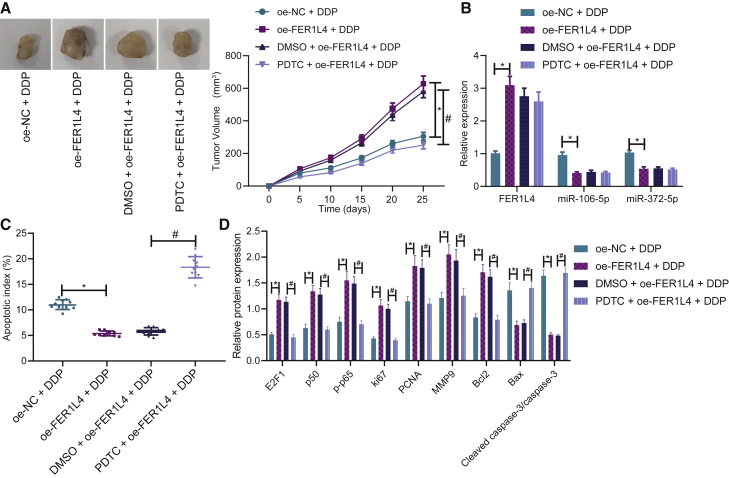
FER1L4 promoted drug-resistant cancer growth that was inhibited by NF-κB inhibitor PDTC (A) Tumor volume. (B) Expressions of miR-106a-5p, miR-372-5p, and FER1L4. (C) Cell apoptosis by TUNEL staining. (D) Expressions of E2F1, p50, p-p65, Bcl-2, Bax, PNCA, Ki67, and MMP9. Data are expressed by the mean ± SD. One-way ANOVA and post hoc Tukey’s test were used for comparisons between multiple groups. Two-way ANOVA was used to compare values at different time points. Repeated measures ANOVA was used for tumor volume comparison at different time points.

Finally, all above results were validated *in vivo* by subcutaneous tumor formation assay in nude mice. Overexpressing FER1L4 increased tumor growth *in vivo* but was reduced by PDTC (Figure 7A). Overexpressing FER1L4 increased the expression of FER1L4 while decreasing the expressions of miR-106a-5p and miR-372-5p (Figure 7B). However, PDTC had no impact on the expressions of FER1L4, miR-106a-5p, and miR-372-5p. Overexpressing FER1L4 reduced apoptosis but was increased by PDTC (Figure 7C). Overexpressing FER1L4 also increased the expressions of E2F1, p50, p-p65, Bcl-2, Ki67, PCNA, and MMP9 protein levels, while reducing the expressions of Bax and cleaved caspase-3 (Figure 7D). These effects were reversed by the addition of PDTC. Hence, FER1L4 promoted tumor growth by increasing the level of drug resistance, while that of which was inhibited by NF-κB inhibitor PDTC.

## Discussion

There are a few important findings in this study. First, the expression of FER1L4 was increased in liver cancer tissues and cells. FER1L4 knockdown inhibited cancer cell proliferation and invasion capabilities but promoted apoptosis. The expressions of miR-106a-5p and miR-372-5p were both decreased in liver cancer, which was associated with a poorer prognosis. FER1L4 knockdown increased the expressions of miR-106-5p and miR-372-5p that inhibited cancer progression. miR-106a-5p and miR-372-5p was bound to E2F1 to reduce cancer progression by inhibiting the expression of E2F1. Overexpressing E2F1 activated the NF-κB pathway, leading to the progression of liver cancer. Lastly, overexpression of FER1L4 levels increased tumor growth *in vivo*, which was inhibited by impeding NF-κB. Collectively, we provided solid evidence, through patient tissues and cell and animal models, that FER1L4 promoted liver cancer by downregulating miR-106a-5p and miR-372-5p, thus leading to an increased expression of E2F1 and NF-κB pathway. These signaling mediators may be potential novel therapeutic targets for the treatment of liver cancer.

The incidence rate of liver cancer in China is increasing at an alarming rate. In 2015, it was estimated that there were 370,000 new cases of liver cancer, equal to approximately 17.64 per 100,000 people.^4^ Liver cancer prevention methods have improved in the recent years, through the better treatment of hepatitis B and C and improved education on the hazardous effects of cigarette smoking.^6^ Despite this, the increasing incidence is multifactorial and may involve drug resistance to treatment.^11^^,^^12^ Therefore, in this study, we sought to understand the pathogenesis of liver cancer using a drug-resistant strain. We found that the expression of FER1L4 was increased, which consequently led to the progression of liver cancer. The results suggested that FER1L4 has oncogenic characteristics that agree with previous studies in gastric cancer and endometrial cancer.^13^^,^^15^. An overview has demonstrated that all FERLIN genes are modulated in several cancer types, among which MYOFERLIN and FER1L4 genes are more frequently upregulated rather than being downregulated.^24^ In addition, based on the databases of The Cancer Genome Atlas and Gene Expression Omnibus, combined with the analysis of breast tumor and adjacent nontumor tissues in the clinical cohort, a previous study has also reported a higher expression of FER1L4 in tumorous tissues, which may be associated with worse disease outcomes and may trigger chemoresistance and tumorigenesis in human cancer.^25^ According to Cox et al.,^14^ FER1L4 expressed at a high level in the tissues of clear-cell renal-cell carcinoma correlated with aspects of tumor aggressiveness, and it also highlighted FER1L4 as an oncogenic driver in human cancers, which may be a potential therapy target. However, some previous studies have showed that FER1L4 may be a tumor suppressor in liver cancer. For example, a previous study suggested that FER1L4 suppressed the proliferation of hepatocellular carcinoma cells through PTEN.^26^ Another study revealed that FER1L4 predicts a good prognosis in liver cancer.^27^ In addition, FER1L4 suppresses liver cancer proliferation and migration through phosphatidylinositol 3-kinase (PI3K)/protein kinase B (AKT) signal pathway.^28^ These discrepancies may be due to different subtypes of cancer cell lines in different studies. It may also be due to the complex function of FER1L4 on the bifunctional impact of FER1L4 in liver cancer, particularly in drug-resistant strains of liver cancer cells.

We then continued to study the downstream signaling molecules of FER1L4 and found that FER1L4 binds and inhibits the expressions of miR-106a-5p and miR-372-5p. This result is similar to other studies in various cancers, including liver cancer.^17^^,^^18^^,^^27^ These results showed that miR-106a-5p has cancer suppressor effects, which has been shown in a previous study.^29^ In addition, miR-106a-5p and miR-372-5p were found to bind to and inhibit E2F1. These results have already been previously shown in other cancers^19^^,^^21^ but appear to be shown for the first time in liver cancer. E2F1 has also been shown previously to increase cancer cell invasion and reduce apoptosis in liver cancer and therefore, has oncogenic activities.^30^, ^31^, ^32^

Another important finding was that E2F1 led to the activation of NF-κB. This result has been shown previously that E2F1 is a transcriptional activator of NF-κB target genes.^22^ More importantly, NF-κB is an important transcription factor that regulates many genes in cancer that are involved in proliferation, migration, and apoptosis,^33^^,^^34^ and the inhibition of NF-κB alleviates tumor growth *in vitro* and *in vivo*. Therefore, NF-κB inhibitor PDTC used in this study may be a potential treatment for liver cancer.

Liver cancer has multiple subtypes and is rendered a highly heterogenous disease. In this study, we used a few liver cancer cell lines, as well as a drug-resistant strain. Some of our results were contrary to previous studies in the literature. Therefore, our results ought to be verified in other types of liver cancers by including more liver cancer cell lines before allowing the findings from this experiment to be applied to all types of liver cancers.

**Figure 8:**
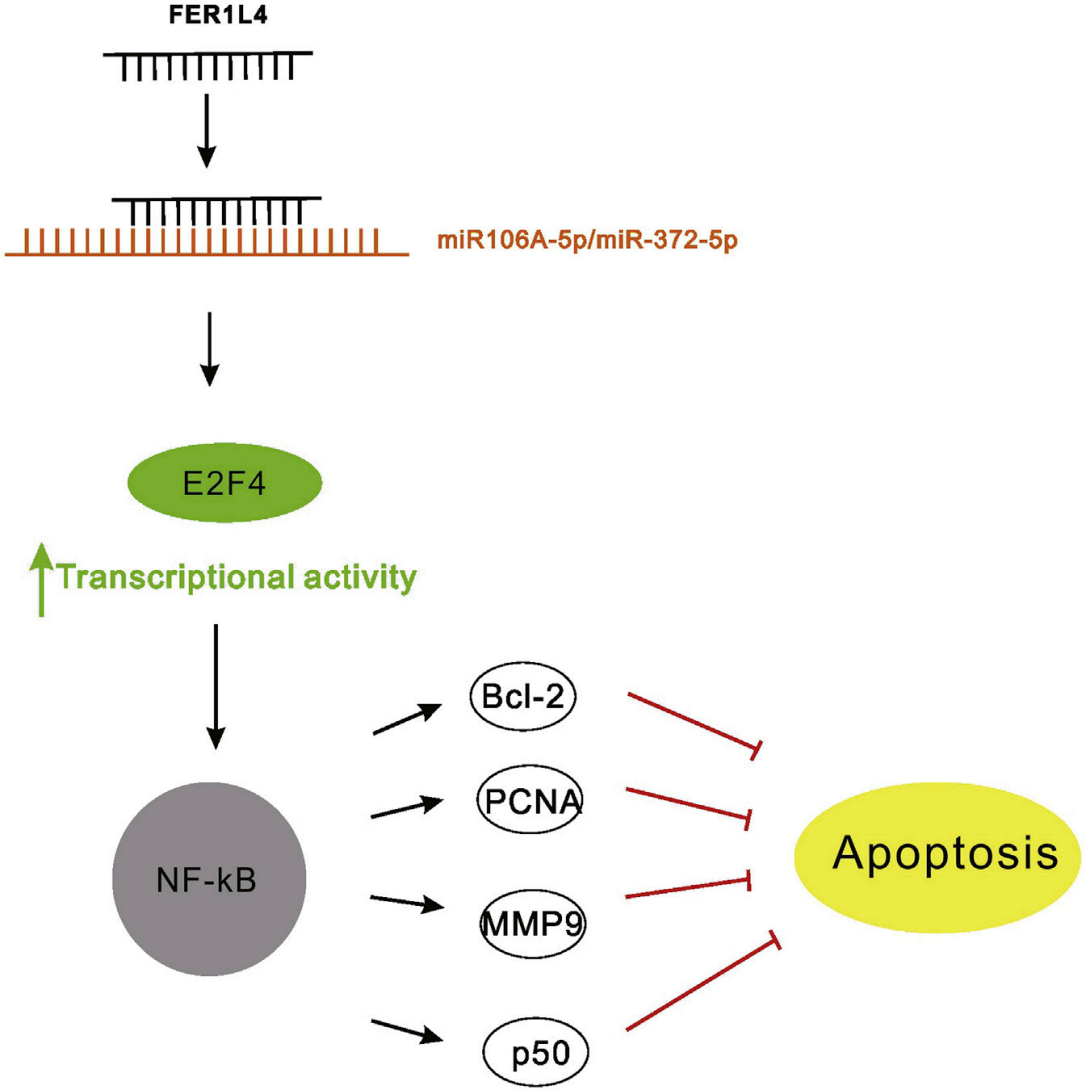
The mechanism diagram of FER1L4/miR-106a-miR-372-5p/E2F1/NF-κB axis in drug-resistant liver cancer FER1L4 inhibits the expressions of miR-106a and miR-372-5p to promote drug-resistant liver cancer progression through the upregulation of E2F1 and the activation of NF-κB.

In conclusion, FER1L4 promotes drug-resistant liver cancer progression through the inhibition of the expressions of miR-106a and miR-372-5p, leading to the upregulation of E2F1 and the activation of NF-κB (Figure 8). The inhibition of NF-κB may be new therapeutic target for the treatment of drug-resistant liver cancer.

## Materials and methods

### Materials

All human and animal procedures were approved by Sichuan Provincial People’s Hospital. 95 patients with liver cancer were enrolled from March 2014 to March 2017. Among them, 49 cases received cisplatin chemotherapy. Specimens used in this study were collected during surgery. Liver cell line THLE-3 and liver cancer cells SK-HEP-1, HEP3B, and HUH-7 were all purchased from the Cell Bank, at the Chinese Academy of Sciences (Shanghai, P.R. China). Human liver cancer cell cisplatin-resistant strain HUH-7/DDP was purchased from Aolu Bio (Shanghai, P.R. China). Female BALB/c nude mice (n = 40, 5-week-old, 18–22 g) were purchased from VitalRiver (Beijing, P.R. China). The animal experiments were approved by Sichuan Provincial People’s Hospital.

### mRNA expression determined by qRT-PCR

Trizol (Cat. No. 16096020, Thermo Fisher Scientific, Waltham, MA, USA) was used to extract total RNA. RNA (5 μg) was reversely transcribed to synthesize complementary DNA (cDNA) by a cDNA kit (K1622; Fermentas, Ontario, CA, USA). TaqMan MicroRNA Assay and TaqMan Universal PCR Master Mix were employed to perform qRT-PCR by using cDNA as a template. PCR reaction was conducted at 95°C for 2 min, followed by 45 cycles at 95°C for 15 s, and 60°C for 45 s. U6 was used as an internal reference.

qRT-PCR was then performed by using TaqMan Gene Expression Assays (Applied Biosystems, Foster City, CA, USA). GAPDH was used as an internal reference. PCR reaction was conducted at 95°C for 10 min, 95°C for 15 s, 60°C for 30 s, and 72°C for 45 s, for 35 cycles in total. All samples were tested in triplicates. Primer sequences are shown in Table 1. Relative mRNA expression was determined by the 2^–ΔΔCt^ methods.

**Table 1:** Primer sequences for qRT-PCR

Name	Sequence
FER1L4-F	5′-ACACAGTCCTTGTGGGTTCC-3′
FER1L4-R	5′-CCTGTCTCCTCCATCTCTCC-3′
E2F1-F	5′-ATGTTTTCCTGTGCCCTGAG-3′
E2F1-R	5′-AGATGATGGTGGTGGTGAC-3′
miR-106-5p-F	5′-GCGGCGGAAAAGTGCTTACAGTG-3′
miR-106-5p-R	5′-ATCCAGTGCAGGGTCCGAGG-3′
miR-372-5p-F	5′-GCGGCGGUCUUAUCACGAGGUTG-3′
miR-372-5p-R	5′-GTGCAGGGTCCGAGGT-3′
U6-F	5′-CATCACCATCAGGAGAGTCG-3′
U6-R	5′-TGACGCTTGCCCACAGCCTT-3′
GAPDH-F	5′-AAGAAGGTGGTGAAGCAGGC-3′
GAPDH-R	5′-TCCACCACCCTGTTGCTGTA-3′

F, forward; R, reverse

### Receiver operating characteristic (ROC) curve analysis

ROC curves were drawn to evaluate the accuracy of gene expression to distinguish the survival rates of patients. The R language pROC package (https://cran.r-project.org/web/packages/pROC/index.html) was used to construct the ROC curve and evaluate survival risk model.

### Protein expression determined by western blotting

Tissues or cells were lysed by using radioimmunoprecipitation assay (RIPA; Beyotime Biotechnology, Shanghai, P.R. China) lysis buffer. The total protein concentration was measured by the Bradford method. Protein concentration was adjusted with 1× PBS. SDS loading buffer (4×) was added and left to heat at 100°C for 10 min. The mixture was centrifuged at 12,000 × *g* for 1 min and the resulting supernatant was collected for loading. Samples were separated using 10% SDS-PAGE at 80 V for 30 min and 120 V for 70 min. Proteins were later transferred onto the polyvinylidene fluoride (PVDF) membrane (Millipore, Burlington, MA, USA). Proteins were left to be blocked by a blocking buffer for 2 h. Membranes were incubated with primary rabbit anti-human E2F1 (ab218527, 1:1,000, Abcam, Cambridge, UK), p50 (ab32360, 1:1,000, Abcam), p-p65 (ab86299, 1:1,000, Abcam), MMP9 (ab38898, 1:1,000, Abcam), PCNA (ab29, 1:1,000, Abcam), Ki67 (ab16667, 1:1,000, Abcam), Bcl-2 (ab32124, 1:1,000, Abcam), Bax (ab32503, 1:1,000, Abcam), and GAPDH (ab128915, 1:1,000, Abcam) antibodies overnight at 4°C. Membranes were washed 3 times with 1× PBST for 10 min each time. Membranes were then incubated with secondary antibodies (ab6785, 1:1,000, Abcam) for 1 h at room temperature. Next, membranes were washed 3 times with 1× PBST for 10 min each time. Enhanced chemiluminescence (ECL; Sangon Biotech, Shanghai, P.R. China) substrate and Tanon 4200 automatic chemiluminescence image analysis system were used for chemiluminescence detection. The gray intensity of each protein band was analyzed by Gel-Pro analyzer.

### Immunohistochemistry

Paraffin sections were heated in an oven at 60°C for 20 min. Sections were then dewaxed for 5 min each in xylene (I, II, III), and 2 min each in absolute ethanol (I, II), 90% ethanol (I, II), 80% ethanol (I, II), and 70% ethanol (I, II). Ethanol was washed by water, and the sections were washed again with PBS for 5 min. After placing sections in the Tris-EDTA buffer solution (pH 9.0), sections were heated in a microwave oven twice for 5 min and allowed to cool to room temperature. Non-specific proteins were left to be blocked with 5% goat serum at room temperature for 30 min. Sections were incubated with primary antibody rabbit anti-human E2F1 (ab218527, 1:1,000, Abcam), p50 (ab32360, 1:1,000, Abcam), p-p65 (ab86299, 1:1,000, Abcam), MMP9 (ab38898, 1:1,000, Abcam), PCNA (ab29, 1:1,000, Abcam), Ki67 (ab16667, 1:1,000, Abcam), Bcl-2 (ab32124, 1:1,000, Abcam), Bax (ab32503, 1:1,000, Abcam) at room temperature for 1 h and then overnight at 4°C. Sections were re-warmed to 37°C for 45 min and incubated with secondary antibodies (ab6712, 1:1,000, Abcam) at 37°C for 30 min. diaminobenzidine (200 μL, Sigma, St. Louis, MO, USA) working solution was added in a dropwise manner to each section, followed by 20 min of incubation in the dark. Counterstaining, dehydration, transparency, and mounting were performed three times with PBS for 3 min each time and then washed with double-distilled water for 5 min. Sections were counterstained with 100 μL of hematoxylin staining solution and then were treated with 80% (I, II), 90% (I, II), and absolute ethanol (I, II) at 2 min each, followed with xylene (I, II) at 5 min each. Lastly, sections were mounted with resin.

### FISH

Paraffin sections were heated in an oven at 60°C for 20 min. Each of the sections was dewaxed for 5 min in xylene (I, II, III) and 2 min in absolute ethanol (I, II), 90% ethanol (I, II), 80% ethanol (I, II), and 70% ethanol (I, II). Sections were treated in deionized water at 90°C for 15 min, in citrate buffer (pH 6.0) for 40 min at 37°C, and proteinase K treatment for 10 min. Sections were then left to fix in methanol at room temperature for 10 min and incubated in 70% ethanol, 85% ethanol, and 100% ethanol at room temperature for 3 min each. Pre-hybridization solution was added and incubated at 42°C for 1 h. Probe hybridization solution with the hybridization probe sequence: 5′-GACCCTGGGCAGCCGGAGAGCTCATTGCCGCCTTTCAACTCATTGAACTAGACTACAGTGGCCGACTTGAGCCCTCAGTGCCCAGTGAGGTGGAGCCCCAGGATCTGGCACCCCTGGTTGAGCCCCACTCTGGACGCCTGTCCCTTCCACCCAACGTGTGCCCAGTGCTCAGGGAGTTCCGTGTTGAGGTGCTGTTCTGGGGTCTTAGGGGACTTGGTCGTGTGCATCTGCTCGAGGTGGAGCAGCCCCAGGTTGTACTGGAGGTGGCTGGGCAAGGTGTGGAGTCTGAGGTCCTGGCCAGCTACCGTGAGAGCCCCAATTTCACTGAGC-3′ was added and incubated at 42°C overnight. Sections were stained with 4′6-diamidino-2-phenylindole, washed with buffer, and imaged under the guidance of a microscope.

### Cell culture and transfection

Normal liver cells THLE-3 and liver cancer cells HUH-7 were cultured in 10% FBS (Gibco, Waltham, MA, USA) and RPMI-1640 (Gibco) medium. Liver cancer cells SK-HEP-1 and HEP3B were cultured in 10% FBS (Gibco) and MEM (Gibco) medium. Human liver cancer cell cisplatin-resistant HUH-7/DDP was cultured in 10% FBS (Gibco), MEM (Gibco) medium, and 20 μM cisplatin at 37°C with 5% CO_2_ in saturated humidity. Transfection was performed with the Lipofectamine 2000 Transfection Reagent (Invitrogen, Carlsbad, CA, USA). Cells at the logarithmic growth phase were seeded at 1 × 10^6^ cells/well in 6-well plates and transfected with the negative control (5′-CAGUACUUUUGUGUAGUACAA-3′), mimic-NC, sh-NC, oe-NC (5′-UUCUCCGAACGUGUCACGUTT-3′), sh-E2F1 (Sh-E2F1-1: 5′-GCCUGGGUGAUUUAUUUAUTT-3′; sh-E2F1-2: 5′-ACCTCTTCGACTGTGACTTTG-3′; sh-E2F1-3: 5′-CGTGGACTCTTCGGAGAACTT-3′), sh-FER1L4 (sh-FER1L4-1: 5′-CAGGACAGCUUCGAGUUAATT-3′; sh-FER1L4-2: 5′-CGTGTACATCTGTTTGAGATA-3′, sh-FER1L4-3: 5′-GCTTCTAGTCAGAGTGTATAT-3′), miR-106a-5p mimic (5′-GAUGGACGUGACAUUCGUGAAAA-3′), or miR-372-5p mimic (sequence: 5′-UCUUAUCACGAGGUGUAAACUCC-3′). Next, 100 μL Opti-MEM was mixed with 50 nM shRNA or miRNA in a 1.5 mL tube. Another 1.5 mL tube was added with 100 μL Opti-MEM and 8 μL Lipofectamine 2000. Two tubes were mixed at room temperature for 20 min. Culture mixture in the 6-well plate was aspired and 900 μL of serum-free culture fluid was added to each well. Transfection mixture was added in a dropwise fashion to a 6-well plate, mixed well and incubated for 5 h. The medium was replaced, and cells were left to culture for 24 or 48 h for qRT-PCR or western blotting.

### Cell proliferation determined by CCK-8

Cells were seeded into 96-well plates at 3 × 10^3^ cells/well and incubated at 37°C in a CO_2_ incubator. 1640/DMEM medium was added to the blank well. Each group was tested in 5 wells. After incubation at the specific time points 0, 24, 48, and 72 h, CCK-8 (Dojindo, Kumamoto, Japan) reaction solution was added to each well and incubated in a CO_2_ incubator for 2.5 h. The absorbance value was determined by a microplate reader at 450 nm.

### Apoptosis determined by flow cytometry

Cells were left to digest with trypsin without EDTA for 2 min and were centrifuged at 1,500 rpm for 5 min at room temperature. Supernatant was discarded and the cell pellet was rinsed twice with PBS to remove pancreatin and medium. Cells were centrifuged again at 1,500 rpm for 5 min and the supernatant was discarded. Cells were resuspended in a 1.5 mL tube with 400 μL 1 × binding buffer (Mbchem, Mumbai, India). Annexin V-fluorescein isothiocyanate (FITC; 5 μL) was added to label apoptotic cells. Cells were incubated at 4°C in total darkness for 15 min and were stained with 10 μL propidium iodide to label the nuclei of apoptotic cells at 4°C in total darkness for 5 min. Cells were detected by flow cytometry (BD FACSCalibur, BD Biosciences, Franklin Lakes, NJ, USA) within a 1 h period.

### Cell invasion determined by Transwell assay

Matrigel was thawed in advance at 4°C and diluted to 50 mg/L with 0.5% FBS in DMEM at a ratio of 1:6. The upper chamber was coated with 60 μL Matrigel and incubated in an incubator for 4 h. Cells in each experimental group were cultured overnight with 5% FBS medium. After trypsin digestion, cells were resuspended in 5% FBS medium and seeded at the upper chamber of the Transwell chamber at 1,000 cells/well. FBS 1640/DMEM culture medium (700 μL, 20%) was added to the lower chamber. Cells were then cultured in a CO_2_ incubator. After cells were cultured for 48 h, observations of those cells were noted at the bottom of the Transwell chamber. Cells were then left to fix in precooled 4% paraformaldehyde at room temperature for 10 min. Those that had not passed through the membrane on the upper chamber were removed by a cotton swab and those at the lower chamber were stained with 500 μL of 0.1% crystal violet at 37°C for 30 min. Cells were imaged and counted under the guidance of a microscope.

### Dual luciferase reporter gene assay

Predicted binding site fragments and mutant fragments of miR-106a-5p, miR-372-5p, and E2F1 were inserted into the dual luciferase reporter vector as reporter plasmids, labeled as E2F1-WT-106a, E2F1-106a-MUT, E2F1-WT-372a, and E2F1-372-MUT. Luciferase reporter vector was co-transfected with miR-106a-5p or miR-372-5p. Transfection was carried out in a 24-well plate, with 10 ng fluorescent reporter carrier and 50 nM miRNA for 6 h. Transfection solution was aspirated and 400 μL of complete culture medium containing 10% FBS were added. Cells were left to continue incubating at 37°C with 5% CO_2_ for 24 h and were collected by using dual luciferase detection kit (Promega E1910, Madison, WI, USA). Cells were lysed with 200 μL of 1× passive lysis buffer. Each sample was tested in triplicate. Luciferase activity was determined by Tecan M1000 (Männedorf, Switzerland). LARII reagent (30 μL) was added to each well. Renilla and Firefly luciferase activities were read by setting the reader at 1–2 s delay and 5–10 s reading.

### RIP assay

RIP kit (Millipore) was used to detect the binding of FER1L4 and miR-106a-5p or miR-372-5p. Cells were lysed with RIPA lysis buffer (P0013B, Beyotime) in an ice bath for 5 min and then centrifuged at 14,000 rpm at 4°C for 10 min. Part of the cell extract was used as the Input and the rest was incubated with antibodies for co-precipitation. The sample and Input were digested with proteinase K to extract RNA for subsequent qRT-PCR analysis. Samples were incubated with rabbit anti-Ago2 (1:100, ab32381, Abcam) at room temperature for 30 min. Rabbit anti-human IgG (1:100, ab109489, Abcam) was used as a negative control. Each experiment was repeated 3 times.

### RNA pull-down

Liver-cancer-resistant strain cells were transfected with transcription-labeled biotin RNA probes (50 nM each). Cells were collected, washed with PBS, and vortexed after being left to transfect for 48 h. Cells were later incubated with a specific cell lysis buffer (Ambion, Austin, TX, USA) for 10 min. The lysate was incubated with M-280 streptavidin magnetic beads (Sigma) and pre-coated with RNase-free and yeast tRNA (Sigma) at 4°C for 3 h. qRT-PCR was used to determine whether miR-106a-5p and miR-372-5p were enriched in the FER1L4 group.

### Subcutaneous tumor formation in nude mice

Human liver cancer cell line cisplatin-resistant strain HUH-7/DDP was left to digest with trypsin at 90% fusion. Single cell suspension was prepared by adding serum-free DMEM. Cells were centrifuged at 1,500 rpm for 3 min and the resulting supernatant was aspirated, followed by the addition with DMEM medium without FBS. Cell concentration was adjusted to 8 × 10^8^ cells/mL. Equal volumes of matrigel (BD) were then added and inoculated subcutaneously in nude mice within 1 h. After 10 days of inoculation, tumor formation was observed with the tumor volume nearing 100 mm^3^ after 14 days. Animals were randomly divided into four groups (n = 10/group): control, Oe-FER1L4 group + DDP group, DMSO + oe-FER1L4 + DDP, and PDTC + oe-FER1L4 + DDP. oe-FER1L4 group was prepared as a transfection complex according to the instructions provided by Lipofectamine 2000. Mice in the oe-FER1L4 group were injected using a sterile syringe 5 mm away from the tumor, subcutaneously. Injection was made in multiple sites (total 50 μL). During the second and fourth injections, cisplatin was administrated twice through the tail vein (Hansoh Pharma, JiangSu, P.R. China). After 2 days of cisplatin administration, all nude mice were euthanized, and the tumors were collected and imaged. Tumors were quickly divided into two parts: half was frozen, and another half was fixed in formalin.

### Apoptosis determined by TUNEL assay

Paraffin sections were heated in an oven at 60°C for 20 min. Sections were dewaxed with 5 min each in xylene (I, II, and III) and 2 min each in absolute ethanol (I and II), 90% ethanol (I and II), 80% ethanol (I and II), and 70% ethanol (I and II). Sections were then washed with ethanol in water and PBS for 5 min. TUNEL test kit (Roche, Basel, Switzerland) was used. Tissues were treated with Proteinase K working solution at room temperature for 20 min and rinsed twice with PBS. TUNEL reaction mixture was prepared by mixing 50 μL terminal deoxynucleotidyl transferase and 450 μL fluorescein dye. Fluorescein-labeled dUTP solution (50 μL) was used as a negative control and DNase I was added and incubated at room temperature for 10 min in advance for the positive controls. After the slides were dried 50 μL TUNEL reaction mixture was added and incubated in complete darkness in a wet box at 37°C for 1 h. After the sections were dried, 50 μL converter-peroxidase was added and incubated in complete darkness in a wet box at 37°C for 30 min. Afterward, cells were incubated with 100 μL diaminobenzidine chromogenic substrate at room temperature for 10 min and counterstained with hematoxylin counterstaining for 2 s.

### Statistical analysis

All statistical analyses were performed using SPSS software version 21.0 (IBM, Armonk, NY, USA). Data are expressed by the mean ± SD. Paired t test was used to compare adjacent tissues with cancer tissues. Unpaired t test was used for the comparison between two groups. One-way analysis of variance (ANOVA) and post hoc Tukey’s test were used for the comparisons between multiple groups. Repeated measures ANOVA was used for tumor volume comparisons at different time points. Two-way ANOVA was used to compare optical density (OD) values at different time points. Pearson correlation was used to analyze the relationship between two factors. Kaplan-Meier method was used to calculate patient survival. Log-rank test was used to perform single-factor analysis. Differences were considered significant when p <0.05.

### Data availability statement

Data sharing not applicable to this article as no datasets were generated or analyzed during the current study.
